# Improving the management of self-harm in primary care

**DOI:** 10.3399/bjgp23X732297

**Published:** 2023-03-30

**Authors:** Faraz Mughal, Liam Clarke, Rachel Connolly, Amanda Yenn Teng Lee, Leah Quinlivan, Nav Kapur

**Affiliations:** GP and National Institute for Health and Care Research (NIHR) Doctoral Fellow, School of Medicine, Keele University, Keele; London Ambulance Service NHS Trust, London; National Institute for Health and Care Excellence (NICE), Manchester; South West London and St George’s Mental Health NHS Trust, London; NIHR Greater Manchester Patient Safety Translational Research Centre, Centre for Mental Health and Safety, University of Manchester, Manchester; Centre for Mental Health and Safety, Manchester Academic Health Sciences Centre, University of Manchester; Greater Manchester Mental Health NHS Foundation Trust; NIHR Greater Manchester Patient Safety Translational Research Centre, University of Manchester, Manchester

Self-harm is sometimes seen by health professionals as a minor problem, yet the risk of suicide is increased fifty-fold in the year after a self-harm episode compared to the general population.^1^ Reducing rates of self-harm is a national policy priority but research suggests that self-harm presentations to general practice are increasing.^2^ Self-harm is defined as intentional self-injury or poisoning regardless of suicidal intent and can occur at any age.^3^ In young females, around one in four have a lifetime history of self-harm.^4^ In older adults who have self-harmed the risk of suicide is particularly high.^5^ Reliable data about self-harm are relatively sparse because self-harm may be hidden, and even when people do present to clinical services self-harm may be poorly recorded.

It is estimated that there are around 228 000 self-harm hospital presentations each year in England that result in NHS treatment costs of ~£128 million.^6^ Even though some people have a single episode of self-harm, around one in five repeat self-harm within 1 year of hospital presentation.^7^ Self-harm is also a global health issue. A few approaches have been tested internationally to reduce repeat self-harm in primary care: neither an educational intervention for GPs targeting older adults or structured GP follow-up reduced repeat self-harm episodes.^8,9^

Within the NHS in the UK, the primary care team is now diverse with new roles such as clinical pharmacists and physician associates supporting the delivery of patient care, but GPs remain pivotal in managing patients who have harmed themselves, and in many cases they may be the senior clinician involved.^10^ Practice nurses describe a lack of confidence managing patients who self-harm.^11^ The primary care setting can facilitate early self-harm identification and intervention in patients to reduce future self-harm behaviour and suicide risk.

In 2022, the National Institute for Health and Care Excellence (NICE) updated its clinical guideline for self-harm: the assessment, management, and preventing recurrence (NG225) supersedes past 2004 and 2011 NICE self-harm guidelines, with several new recommendations for primary care.^3^ The guidance is applicable to all people who have self-harmed except people with repetitive stereotypical self-injurious behaviour such as head-banging. These new primary care recommendations offer an opportunity to improve the standard and quality of care given to patients who have self-harmed.

## How Should GPs Assess People After Self-Harm?

The guideline outlines how a patient who has self-harmed should be treated with *‘respect, dignity and compassion, with an awareness of cultural sensitivity’*.^3^ Clinicians should *‘establish the means of self-harm and, if accessible to the person, discuss removing this with therapeutic collaboration or negotiation, to keep the person safe’*.^3^ GPs should establish the severity of any injury, mental state, and need for further specialist input.

The guideline states that for patients managed in primary care the assessing GP should ensure the person receives regular GP appointments for review of self-harm, information about available support, care for coexisting mental health problems, and a medicines review. Patients who self-harm value continuity of GP care.^12^

It is recommended that GPs consider referring patients who have self-harmed to mental health services for a comprehensive psychosocial assessment. Box 1 outlines when a referral to mental health professionals is recommended as a priority.

The guideline does not recommend the use of risk assessment tools or scales and risk stratification into low, medium, or high for future self-harm or death by suicide, after an episode of self-harm. The pooled estimate of positive predictive values (patients who were scored at ‘high’ risk of suicide and went on to die by suicide) of all risk scales/tools on future death by suicide is 6%.^13^ Instead, GPs should focus *‘on the person’s needs and how to support their immediate and long-term psychological and physical safety’*,^3^ which can be done across consultations. Identifying protective factors can give hope to the patient, which may improve their sense of belongingness and self-worth.

## How Can GPs Intervene?

GPs should provide patients with information regarding their care pathway, and it is recommended that GPs reach a shared decision with the patient (and family/carers where applicable) about the *‘purpose, format and frequency of initial aftercare’*,^3^ and clearly document this. This person-centred care can reduce distress and hopelessness, and empower people who have harmed themselves.^12^ There is guidance on providing aftercare *‘within 48 hours of the psychosocial assessment’* when there are ongoing safety concerns for the person, because repeat self-harm is most likely to occur 2–3 days after the last episode.^3^

The guideline recommends that adults who self-harm are offered astructured and tailored cognitive behavioural therapy-informed psychological intervention (at least four sessions) because there is evidence of positive effects on repetition of self-harm and on hopelessness and depression at post-intervention assessment.^3,14^ For children and young people with significant emotional dysregulation difficulties who have frequent episodes of self-harm, dialectical behavioural therapy for adolescents should be considered.

Accessing appropriate aftercare for patients following self-harm can be challenging. Waiting times are often long and exclusion from mental health services is unfortunately commonly reported for patients who have self-harmed.^15^ However, there is significant opportunity to collaboratively develop accessible self-harm interventions and services(outlined in the *NHS Long Term Plan*), which improve integration between primary and secondary care while learning from examples of best practice. For instance, teams have developed brief intervention follow-up services to facilitate better transitions between primary and secondary care (Step Forward Psychological Intervention Service in Liverpool), and outpatient clinics in liaison psychiatry teams. Future interventions should be developed through engagement with the Talking Therapies programme, liaison psychiatry services, community mental health teams, the voluntary sector, patients and the public, and GPs in practice.

GPs are to consider developing a safety plan with patients after self-harm: there is guidance on how safety plans should be used, what to include, and how to ensure they are kept up-to-date and accessible by the patient, family/carers, and other professionals where appropriate. The guideline covers the use of harm minimisation strategies, including circumstances where they should be considered as an adjunct to other treatment, and which strategies might be useful for people who have self-harmed.

## Safer Prescribing

Primary care prescribers should consider *‘the toxicity of the prescribed medications for people at risk of overdose (for example, opiate-containing painkillers and tricyclic antidepressants) …* [and] *recreational drug and alcohol consumption, the risk of misuse, and possible interactions with prescribed medicines’.*^3^ Teams should consider undertaking a medicines review after self-harm considering toxicity risk and concurrent use of medicines such as benzodiazepines and opiates, and discuss limiting medicines quantities with patients with a history of self-poisoning: alerts on patient records can support this.

## Implementation

Nearly two decades after the first NICE clinical guideline on self-harm, these new primary care recommendations are welcomed and should lead to improved care for this vulnerable group of patients. Though, within the current extremely pressured healthcare environment, there are substantial barriers for GPs to act on the recommendations: lack of time, challenges ensuring continuity of GP care, and difficulties accessing local mental health specialist care are all too familiar to colleagues working on the frontline. To facilitate the adoption of these recommendations, it’s vital to invest in accessible local self-harm services that support primary care networks, evidence-informed staff training, and growing access to recommended psychological therapies.

It is important to note that the guideline is not suggesting that GPs assume the role of specialist mental health professionals. Neither are they suggesting that societal drivers of self-harm such as economic adversity or online safety are amenable to clinical intervention; however, managing self-harm is a daily reality for many in primary care, and is something that should be within the core competency of every GP.

## Box 1 Recommendations for when to make an urgent referral to a mental health specialist

- The person’s levels of concern or distress are rising, high, or sustained.
- The frequency or degree of self-harm or suicidal intent is increasing.
- The person providing assessment in primary care is concerned.
- The person asksfor further support from mental health services.
- Levels of distress in family members or carers of children, young people, and adults are rising, high, or sustained, despite attempts to help.
